# Serologic Status and Toxic Effects of the SARS-CoV-2 BNT162b2 Vaccine in Patients Undergoing Treatment for Cancer

**DOI:** 10.1001/jamaoncol.2021.2675

**Published:** 2021-07-08

**Authors:** Tal Goshen-Lago, Ithai Waldhorn, Roy Holland, Moran Szwarcwort-Cohen, Anat Reiner-Benaim, Yael Shachor-Meyouhas, Khetam Hussein, Liana Fahoum, Mali Baruch, Avivit Peer, Yoram Reiter, Ronit Almog, Michael Halberthal, Irit Ben-Aharon

**Affiliations:** 1 Division of Oncology, Rambam Health Care Campus, Haifa, Israel; 2 Virology Laboratory, Rambam Health Care Campus, Haifa, Israel; 3 Clinical Epidemiology Unit, Rambam Health Care Campus, Haifa, Israel; 4 General Management, Rambam Health Care Campus, Haifa, Israel; 5 Rappaport Faculty of Medicine, Technion, Haifa, Israel; 6 Technion-Integrated Cancer Center, Technion, Haifa, Israel

## Abstract

**Importance:**

The efficacy and safety profile of SARS-CoV-2 vaccines have been acquired from phase 3 studies; however, patients with cancer were not represented in these trials. Owing to the recommendation to prioritize high-risk populations for vaccination, further data are warranted.

**Objective:**

To evaluate the use and safety of the BNT162b2 vaccine in patients undergoing treatment for cancer.

**Design, Setting, and Participants:**

In January 2021, mass SARS-CoV-2 vaccination of high-risk populations, including patients with cancer, was initiated in Israel. This cohort study prospectively enrolled and followed up patients with cancer and healthy participants between January 15 and March 14, 2021. The study was conducted at the Division of Oncology of Rambam Health Care Campus, the major tertiary (referral) medical center of northern Israel. Participants included 232 patients with cancer who were receiving active treatment after the first and second doses of the BNT162b2 vaccine and 261 healthy, age-matched health care workers who served as controls.

**Exposures:**

Serum samples were collected after each vaccine dose and in cases of seronegativity. Questionnaires regarding sociodemographic characteristics and adverse reactions were administered at serum collection. A regulatory agencies–approved assay was used to assess IgG at all time points. Patients’ electronic medical records were reviewed for documentation of COVID-19 infection and results of blood cell counts, liver enzyme levels, and imaging studies.

**Main Outcomes and Measures:**

Seroconversion rate after the first and second doses of the BNT162b2 vaccine and documented COVID-19 infection.

**Results:**

Of the 232 patients undergoing treatment for cancer, 132 were men (57%); mean (SD) age was 66 (12.09) years. After the first dose of BNT162b2 vaccine, 29% (n = 25) patients were seropositive compared with 84% (n = 220) of the controls (*P* < .001). After the second dose, the seropositive rate reached 86% (n = 187) in the patients. Testing rate ratios per 1000 person-days after the first dose were 12.5 (95% CI, 3.4-45.7) for the patients and 48.5 (95% CI, 37.2-63.2) for the controls. Patients undergoing chemotherapy showed reduced immunogenicity (odds ratio, 0.41; 95% CI, 0.17-0.98). In seronegative patients, the rate of documented absolute leukopenia reached 39%. No COVID-19 cases were documented throughout the study period; however, 2 cases in the patient cohort were noted immediately after the first dose. Reported adverse events were similar to data in former trials comprising mostly healthy individuals.

**Conclusions and Relevance:**

In this cohort study, the SARS-CoV-2 BNT162b2 vaccine appeared to be safe and achieve satisfactory serologic status in patients with cancer. There was a pronounced lag in antibody production compared with the rate in noncancer controls; however, seroconversion occurred in most patients after the second dose. Future real-world data are warranted to determine the long-term efficacy of the vaccine with regard to type of anticancer treatment.

## Key Points

**Question:**

What is the serologic status and incidence of adverse effects in patients with cancer who are receiving therapy after administration of the SARS-CoV-2 BNT162b2 vaccine?

**Findings:**

This cohort study evaluated serologic status and safety of the BNT162b2 vaccine in 232 patients receiving active treatment for cancer and 261 health care workers who served as controls. After the first dose of the vaccine, 29% of the patients were seropositive compared with 84% of the controls; after the second dose, the seropositive rate of the patients reached 86%, and reported adverse events resembled those of healthy individuals.

**Meaning:**

The SARS-CoV-2 BNT162b2 vaccine appears to be safe with satisfactory levels of seropositivity in patients undergoing treatment for cancer, although protection may occur later compared with the healthy population.

## Teaser

This cohort study examines the serologic status of patients receiving active treatment for cancer after administration of the SARS-CoV-2 BNT162b2 vaccine.

## Introduction

The COVID-19 pandemic has been associated with inferior clinical outcomes in patients with cancer owing to altered delivery of care and potential high-risk for SARS-CoV-2 infection in distinct subpopulations. Studies have indicated that patients with cancer may experience a severe COVID-19 clinical course, with metastatic cancer, hematologic malignant neoplasms, and lung cancer recognized as leading risk factors.^1,2^ Nevertheless, other studies reported that patients with cancer did not appear to have additional risk compared with the general population and, moreover, that COVID-19 severity was associated with patient age and cardiovascular comorbidities, which are prominent general risk factors in patients with COVID-19.^3^ The association between the pandemic and care delivery appears less controversial. Routine clinical care has been disrupted throughout an extended period while health care clinicians and professional organizations continuously modified recommendations based on risk-benefit assessment.^4^ Owing to the need to alleviate the magnitude of the pandemic and move toward a return to normalcy, effective prophylactic vaccines have been developed and studied in an expedited process.

The BNT162b2 vaccine, which is administered in 2 doses at a 21-day interval, was found to be safe and efficient in preventing COVID-19 in the general population. The phase 2/3 part of the BNT162b2 vaccine global phase 1/2/3 trial served as a basis for its authorization and application worldwide. Although cancer had not been an exclusion criterion in the trials, use of cytotoxic therapy or systemic corticosteroids throughout the study were considered as such in the pioneer study.^5^ In December 2020, the SARS-CoV-2 BNT162b2 vaccine was approved in Israel followed by an extensive mass immunization operation ranking health care workers first followed by high-risk populations, as suggested worldwide.^6^

Polack and colleagues^5^ reported an early vaccine efficacy level in preventing COVID-19 of 52.4% before dose 2 and 90.5% on days 2 to 7 after the second dose; real-world data obtained retrospectively in Israel further substantiated the results of the initial prospective study. Recently, in a large-scale study, data from Israel's largest health care organization were used to appraise the BNT162b2 messenger RNA (mRNA) vaccine efficacy, reflected by documented COVID-19 infection–related hospitalization and mortality rates in a newly vaccinated cohort matched and compared with unvaccinated controls according to demographic and clinical characteristics.^7^ Each cohort consisted of 596 618 persons. Estimated vaccine effectiveness during the follow-up period starting 7 days after the second dose was 92% for documented infection, 94% for symptomatic COVID-19, 87% for hospitalization, and 92% for severe COVID-19. Among the study population, 1.9% of the vaccinated cohort and 2% of the unvaccinated control had a diagnosis of cancer. Another study indicated that, in a cohort of 7214 health care workers, the adjusted rate reductions of SARS-CoV-2 infections were 30% (95% CI, 2%-50%) on days 1 to 14 and 75% (95% CI, 72%-84%) on days 15 to 28 after vaccination.^8^ Although real-world data regarding vaccine effectiveness are emerging depicting a positive outcome in the general population, there is a paucity of data regarding the efficacy and safety in the population of patients with cancer. Despite a lack of evidence on clinical outcomes of the SARS-CoV-2 vaccines in patients with cancer, several professional organizations had established recommendations for vaccination of patients with cancer. Guidelines encompass all patient populations with cancer, regardless of treatment type or underlying cancer.^9^

In this study, we prospectively evaluated the serologic status and safety of the SARS-CoV-2 BNT162b2 vaccine in a cohort of patients with solid tumors who were receiving active anticancer treatments compared with age-matched health care workers who served as vaccinated controls.

## Methods

### Participants and Design

This cohort study included patients with solid tumors receiving intravenous treatment administered at the infusional ambulatory unit of the oncology center or inpatient service within the Rambam Health Care Campus (RHCC), Haifa, Israel (hematologic malignant neoplasms are treated in a separated institution and hence were not included in the study population). As mass vaccination of high-risk populations was launched in Israel from January 10, 2021, patients with cancer without documented COVID-19 infection who were vaccinated (first dose and/or second dose) were randomly enrolled during their routine visit to the oncology center. The first set of data was collected before the second dose (>10 days after first vaccine dose) and the second set was collected about 14 days after the second vaccination. If serologic test results were negative after the second dose of the vaccine, an additional blood sampling was performed 4 weeks after the second dose. If a patient refused a second test, we included only data from the first test in the analysis. The control group consisted of healthy health care workers who underwent serologic testing before the second vaccination dose. At RHCC, all health care workers who were willing to participate in the study underwent serologic testing before the second dose. Once the accrual of patients into the present study was completed and the cohort profile was established, an age-matched cohort was randomly cropped (computer-generated) from the larger general control cohort to match the same age range of the patients and avoid selection bias.

All patients agreed to participate and signed an informed consent form; no financial compensation was provided. If results of the initial serologic test were positive, no additional test was performed. The study protocol was approved by the institutional ethics committee of RHCC. This study followed the Strengthening the Reporting of Observational Studies in Epidemiology (STROBE) reporting guideline. Electronic health records of RHCC were accessed by the study investigators to review patients’ clinical characteristics as well as laboratory test results (complete blood cell count, liver enzyme levels, and creatinine levels) and imaging assays (positron emission tomography and computed tomography scans) performed as part of routine cancer care (January 15 to March 14, 2021) as well as documented COVID-19 infection (determined using reverse transcriptase polymerase chain reaction assay) throughout the study period.

Serum samples were analyzed at all measurement times for the detection of anti–SARS-CoV-2 antibodies. For IgG expression, we used SARS-CoV-2 anti-spike (S) S1/S2 IgG assay (Liaison; DiaSorin) to detect S1/S2 IgG antibodies. Cutoff values for positive serologic findings were 15 arbitrary units per milliliter, as previously established.^10^ All serologic tests were conducted at the RHCC Virology Diagnostic laboratory.

### Statistical Analysis

Negative and positive serologic samples among patients with cancer and controls were compared using the χ^2^ test or Fisher exact test for categorical variables and a 2-tailed unpaired *t* test for age. Adjusted odds ratios were calculated using multivariate logistic regression with a stepwise model-reduction procedure, including the covariates of sex, age, type of treatment, disease stage, laboratory tests, imaging assays, and reported adverse events. Rate ratios per 1000 person-days were calculated and adjusted using Poisson regression, with the logarithm of days from vaccine to serologic test as an offset. Statistical analysis was conducted using R, version 4.1.0 (R Foundation for Statistical Computing). The significance threshold was set at *P* < .05 for the 2-sided unpaired tests.

## Results

### Participants

The study included 232 patients with solid tumors who were receiving active intravenous treatment at the RHCC oncology center and a cohort of 261 aged-matched health care workers from RHCC who served as controls. The patient group comprised 132 men (57%) and 100 women (43%) (mean [SD] age, 66 [12.09] years; median age, 68 years; range, 25-88); the control group comprised 143 women (55%) and 118 men (45%) (mean [SD] age, 59 [15.7] years; median age, 64 years; range, 25-81). In the patient group, 86 patients were tested after the first vaccination dose and 218 were tested after the second vaccination dose (74 patients were tested after both doses). All participants were randomly recruited to the study. Adherence to testing after the first dose was lower than after the second dose; patients preferred to be tested at the latter point.

Patient characteristics are reported in Table 1. Most patients (172 [74%]) had metastatic disease. The most common cancers were gastrointestinal (63 [27%]), genitourinary (48 [21%]), lung (45 [19%]), and breast (42 [18%]). Treatment protocols consisted of chemotherapy (134 [58%]), biological agents (81 [35%]), and immunotherapy (83 [36%]), and some patients received more than 1 treatment class.

**Table 1.** Patient Characteristics

Characteristic	No. (%)
Total	232 (100)
Age, median (range), y	68 (25-88)
Sex	
Female	100 (43)
Male	132 (57)
Type of cancer	
Gastrointestinal	63 (27)
Breast	42 (18)
Genitourinary	48 (21)
Gynecologic	11 (5)
Head and neck	11 (5)
Lung	45 (19)
Melanoma	5 (2)
Neurologic	5 (2)
Sarcoma	2 (1)
Stage	
Local	60 (26)
Metastatic	172 (74)
Treatment	
Chemotherapy	134 (58)
Biological agent	81 (35)
Immunotherapy	83 (36)

### SARS-CoV-2 Serologic Status and Infection

We categorized the cohorts by age as demonstrated in Table 2. In participants aged 60 years or older, only 30% (n = 26) of the patients with cancer had positive serologic test results compared with 80% (n = 146) of the control group (*P* < .001). Nonetheless, for the seropositive patients, the titer score (median, 40.25) did not differ significantly from the titer score of the seropositive controls (median, 43.5). In the group younger than 60 years, 27% (n = 7) of the patients had positive serologic test results (median, 72.5) compared with 94% (n = 94) of the controls (median, 74.0) (*P* < .001). Analysis by age, sex, or disease stage yielded no significant differences within the patient cohort, as depicted in Table 3. In addition, no significant difference was detected in days after vaccine administration for serologic analysis between patients with positive (median, 19 days) vs negative (median, 17 days) serologic findings (*P* = .42). An adjusted Poisson regression model of positive serologic testing rate ratio per 1000 person-days revealed that 12.5 patients would be expected to have positive serologic findings after the first vaccine dose (*P* < .001; 95% CI, 3.4-45.7) compared with 48.5 participants from the control group (*P* < .001; 95% CI, 37.2-63.2). Furthermore, a separate χ^2^ test used to compare the incidence rates between the groups resulted in a significant difference (*P* < .001) of 84.3% (220 of 261) among the control group compared with 26.5% (26 of 98) among the patients being treated for cancer. Table 4 summarizes patient characteristics after the second vaccine dose.

**Table 2.** Serology Results After First Vaccine Dose

Serologic status	Cancer group, No. (%)			Control group, No. (%)		
	Age <60 y	Age ≥60 y	All patients	Age <60 y	Age ≥60 y	All controls
Total, No. (%)	26 (30)	60 (70)	86 (100)	79 (30)	182 (70)	261 (100)
Seropositive, No. (%)	7 (27)	18 (30)	25 (29)	74 (94)	146 (80)	220 (84)
Titer score, median	72.5	40.25	42.3	74.0	43.5	72.0

**Table 3.** Patient Characteristics After First Vaccine Dose

Characteristic	Patients after first vaccine dose, No. (%)		
	Positive serologic results	Negative serologic results	All
Total	25 (29)	61 (71)	86 (100)
Median day from vaccine dose	19 (13-21)	17 (10-30)	17 (10-30)
Sex			
Female	11 (44)	23 (38)	34 (40)
Male	14 (56)	38 (62)	52 (60)
Age, median (range), y	70 (53-86)	67 (32-86)	68 (32-86)
Treatment			
Chemotherapy	15 (60)	35 (57)	50 (58)
Biological agent	9 (36)	20 (33)	29 (34)
Immunotherapy	10 (40)	24 (39)	34 (40)
Type of cancer			
Breast	5 (20)	13 (15)	18 (21)
Prostate	4 (16)	1 (1)	5 (6)
Lung	3 (12)	11 (13)	14 (16)
Colorectal	2 (8)	6 (7)	8 (9)
Melanoma	2 (8)	0	2 (2)
Neurologic	1 (4)	0	1 (1)
Bladder	2 (8)	5 (6)	7 (8)
Gynecologic	2 (8)	4 (5)	6 (7)
Kidney	1 (4)	6 (7)	7 (8)
Head and neck	1 (4)	5 (6)	6 (7)
Hepatobiliary	2 (8)	4 (5)	6 (7)
Esophagus and gastric	0	2 (2)	2 (2)
Pancreas	0	4 (5)	4 (5)
Stage			
Local	6 (24)	20 (33)	26 (30)
Metastatic	19 (7)	41 (67)	60 (70)

**Table 4.** Patient Characteristics After Second Vaccine Dose

Characteristic	Patients after second vaccine dose, No. (%)		
	Positive serologic results	Negative serologic results	All
Total	187 (86)	31 (14)	218 (100)
Median day from vaccine dose (range)	15 (2-54)	20 (5-48)	16 (2-54)
Sex			
Female	79 (42)	13 (42)	92 (42)
Male	108 (58)	18 (58)	126 (58)
Age, median (range), y	68 (35-88)	68 (25-84)	68 (25-88)
Treatment			
Chemotherapy	102 (55)	23 (74)	125 (57)
Biological agent	70 (37)	7 (23)	77 (35)
Immunotherapy	71 (38)	8 (26)	79 (36)
Type of cancer			
Lung	37 (20)	6 (19)	43 (20)
Colorectal	30 (16)	4 (13)	34 (16)
Breast	29 (16)	9 (29)	38 (17)
Kidney	14 (7)	3 (10)	17 (8)
Prostate	14 (7)	1 (3)	15 (7)
Bladder	12 (6)	1 (3)	13 (6)
Hepatobiliary	10 (5)	0	10 (5)
Head and neck	10 (5)	1 (3)	11 (5)
Esophagus and gastric	3 (2)	2 (6)	5 (2)
Pancreas	9 (5)	2 (6)	11 (5)
Gynecological	8 (4)	1 (3)	9 (4)
Neurological	5 (3)	0	5 (2)
Melanoma	4 (2)	0	4 (2)
Testis	1 (1)	0	1 (0.5)
Sarcoma	1 (1)	1	2 (1)
Stage			
Local	43 (23)	10 (32)	53 (24)
Metastatic	144 (77)	21 (68)	165 (76)

Review of the electronic medical records noted that 2 cases of COVID-19 infection were documented after the first dose in the patient cohort. Otherwise, there were no documented cases of COVID-19 in either cohort throughout the study period.

### Local and Systemic Reactions

Pain at the injection site within hours to several days after an injection was the most commonly reported local reaction (69%); lower percentages of patients reported injection-site warmness (9%), redness (8%), or swelling (4%) (Figure, A). The most commonly reported systemic reactions were fatigue (24%), muscle and joint pain (13%), and headache (10%); 1% of the patients reported a fever event (temperature, ≥38 °C) (Figure, B). Elevation of liver enzyme levels (alanine aminotransferase, aspartate aminotransferase, alkaline phosphatase, and γ-glutamyltransferase) of more than 1.5 times from baseline levels was documented in 24 patients up to 6 weeks after the first vaccine dose. Newly documented regional lymphadenopathy (cervical or axillary) was noted in 5% of computed tomography or positron emission tomography scans (performed as routine cancer care) throughout the study period.

**Figure.**
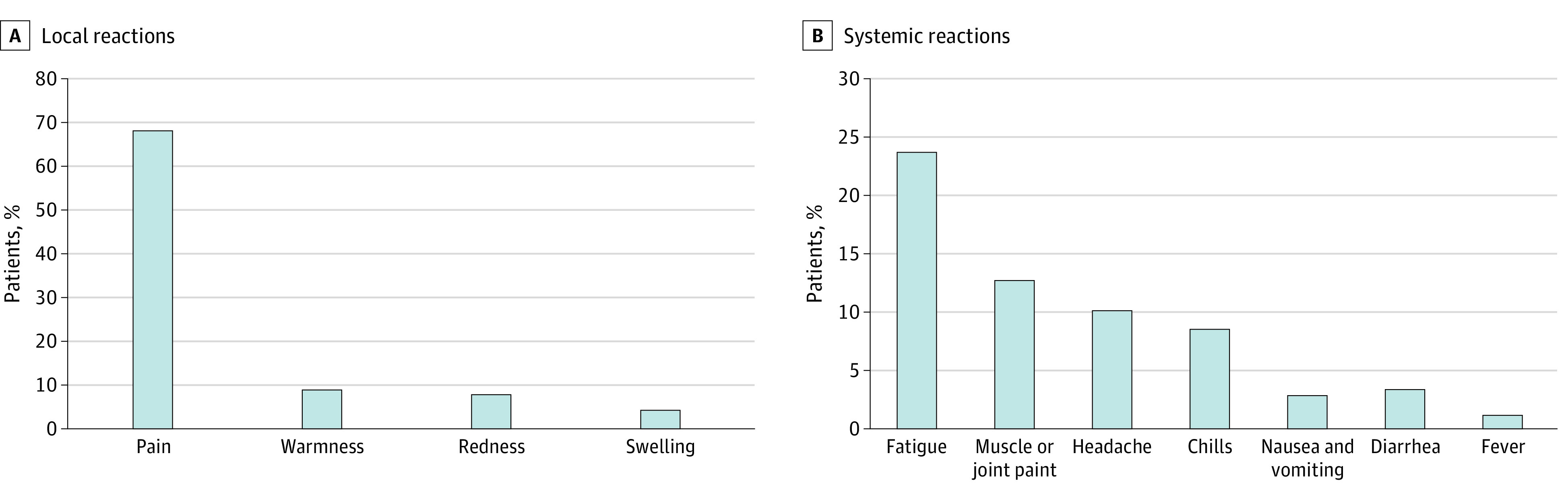
Summary of Local and Systemic Reactions Reported by Patients

## Discussion

Despite evolving evidence on the efficacy and safety of SARS-CoV-2 vaccines, there is a paucity of data on patients with cancer who are receiving active anticancer therapy. The BNT162b2 vaccine was approved by regulatory agencies based on a phase 2/3 trial that randomized a total of 43 548 participants to receive 2 doses of the vaccine or a control agent at a 21-day interval.^5^ Subgroup analysis revealed similar efficacy across age, sex, and comorbidities that were risk factors for severe manifestation of COVID-19. Among included patients, cancer had not been considered as an exclusion criterion. In the list of comorbidities, “any malignancy” was documented in 1395 (3.7%) patients in both study groups. Of those participants, 1 case of COVID-19 in the vaccine group and 4 cases in the control group yielded a 75.5% efficacy rate for the vaccine. Moreover, in the Moderna COVID-19 vaccine, the mRNA-1273, which had also been approved by the regulatory agencies based on an ongoing phase 3 trial evaluating the vaccine efficacy,^11^ study exclusion criteria included patients who had received systemic cytotoxic therapy or immune-modifying drugs for more than 14 days in total within 6 months before screening. Taken together, current data regarding the 2 vaccines do not represent patients with cancer who are receiving active cancer therapy.

To our knowledge, this study is the first to prospectively characterize the serologic status, immunogenicity, and safety of the BNT162b2 vaccine in a cohort of patients with solid tumors who are receiving active anticancer therapies. The study was conducted at the Division of Oncology of RHCC, the major tertiary (referral) medical center of northern Israel, which serves a heterogeneous population that represents patients with cancer throughout the country. Patients were enrolled in the study, preferably after the first dose of the vaccine, and followed up for 2 months. Health care workers who were age-matched served as vaccinated controls. Immunogenicity after the first dose and before the second dose reflected by positive serologic findings revealed significantly lower rates in patients with cancer compared with controls; age was not a confounder. Seroconversion occurred at higher rates in the patient cohort after the second dose and reached positivity in 86% (n = 187) of the patients. Two cases of COVID-19 infection were documented immediately after the first dose among the vaccinated patients. The safety profile appeared to be in concordance with previous reports,^5,11^ suggesting that the vaccine is not associated with a higher rate of adverse events in patients with cancer.

However, because these patients are routinely monitored through biochemical studies, complete blood cell counts, and imaging studies as part of their cancer care, we noted an increase in liver enzyme levels in 10.3% of the patients (spontaneously resolved in 37% of the patients), and in 5% regional lymphadenopathy (cervical or axillary) was depicted in computed tomographic or positron emission tomographic scans that had not been documented in earlier examinations. It has previously been shown in vaccination studies of other viruses that temporary redistribution of lymphocytes from the systemic circulation to lymphoid tissues may be induced by immune stimulation produced by the vaccine.^12,13^ Patients with cancer demonstrated a gradual, slower immunogenicity compared with the general population.^14^ Although the study population comprised older individuals, we could not detect an age-dependent pattern in the patient cohort, but this change was evident in the control group, which is in accordance with former evidence of an age-dependent manner of immune response to vaccines.^15,16,17^

Fourteen percent of the patients were seronegative at the latest time point (4 weeks after the second vaccine). Patients with breast cancer comprised 29% of the seronegative group, and 74% of these patients were treated with chemotherapy, and the treatments were diverse. Hence, we cannot assume that a specific class of drugs may hamper immunogenicity but rather that lymphosuppressive agents may induce a lack of effective seroconversion. Former indication regarding the role of humoral and cellular immunity in the mechanism of host response to COVID-19 is unclear. A strong, cell-mediated immune response entailing helper T1 cell–biased CD4^+^ and CD8^+^ lymphocytes elicited by the BNT162b1 vaccine has been reported, yet the cellular mechanism remains to be elucidated.^18^ Findings from implementation of other vaccines in immunosuppressed populations indicated that inactivated influenza vaccines are safe in immunocompromised patients, although a trend toward an impaired humoral vaccine was observed. To our knowledge, there are no data regarding mRNA-based vaccines in this population.^14^ Moreover, 1 study showed that antibody titers observed in a cohort of patients with cancer after influenza vaccination (71%) were lower than those in healthy controls (94%).^19^ It is noteworthy that, although in the setting of other vaccines neutralizing antibody levels had been associated with vaccine effectiveness, this association has not been confirmed in the setting of SARS-CoV-2 vaccines; however, association was shown in animal models.^20,21^

### Limitations

There are several limitations to our study. First, the study cohort represents an older population, in which the median age was 68 years. An immune response to many other vaccines had been previously documented to be reciprocally associated with age.^22^ Nevertheless, in the Moderna mRNA-1273 vaccine study, titers to 614D on the pseudovirus neutralization assay were, at an early point, undetectable and demonstrated dose-dependent responses that were observed as early as 7 days after the second dose (day 36) in an age-independent manner.^23^ Second, patients with cancer tend to be cautious and adherent to recommended safety measures in their surrounding environment, such as social distancing and applying a facial mask, along with vaccination throughout the study period, which may affect the infection rate regardless of their vaccination effect. The lack of a control group of patients who were not vaccinated and screened for COVID-19 infection may be a study limitation. In addition, because the study design did not include measurement of a baseline serologic titer before the first vaccine dose, we cannot exclude the possibility that the high seropositivity rate among controls may be attributed to former viral exposure. However, in a study conducted in a representative cohort of health care workers evaluating asymptomatic exposure to COVID-19 throughout the pandemic, using the same antibody detection assay, only 2 of 107 health care workers were found to have a positive anti–SARS-CoV-2 IgG titer, ruling out a substantial rate of former seropositivity.^24^ Third, the lack of systematic registration in the electronic health records of all corticosteroids (oral or intravenous) that may be used by the patients that may affect antibody production represents a study limitation. Fourth, the role of a serologic titer as a sole surrogate biomarker for immunogenicity remains to be elucidated.

## Conclusions

This cohort study provides data on use of the SARS-CoV-2 BNT162b2 vaccine in patients with solid tumors who are receiving active anticancer treatments in the real-world setting. Although the immunogenicity pattern was gradual and slower than in the noncancer population, after the second dose most patients were seropositive and no documented cases of COVID-19 infection were determined. Our study lends credence to the widely adopted recommendation to prioritize patients with cancer for SARS-CoV-2 vaccination. Nevertheless, our results imply that a potential intention to decline a second vaccine by some jurisdictions owing to a shortage of vaccines warrants reevaluation of unique populations, such as patients with cancer, in view of lagging immunogenicity. Until additional prospective data regarding vaccine efficacy in patients with cancer are established, adherence to risk reduction health care strategies is prudent.
